# Supplementary material for: Foreign Healthcare Professionals in Germany: A Questionnaire Survey Evaluating Discrimination Experiences and Equal Treatment at Two Large University Hospitals
Source: Healthcare (Basel). 2022 Nov 22;10(12):2339. doi: 10.3390/healthcare10122339 (PMC9777572; doi:10.3390/healthcare10122339)
Supplement: Supplementary file 1 [file healthcare-10-02339-s001.zip › healthcare-1989662 supplementary.pdf]

Quantitative survey on the working environment/integration/professional qualification of Charité / UKE employees born outside of Germany

Consent *Link: Deutsche Version*

Dear colleague,

Thank you very much for participating in our study!

This study aims to assess the working and living conditions of migrant health care workers.
By identifying possible difficulties and disadvantages, we aim to create a basis for support structures and measures against discrimination.

The survey is aimed at employees of the Charité / UKE **born outside of Germany**.

Participation in the survey is **voluntary** and takes about ***30-45 minutes***.

No labour law consequences can be derived from your answers.

The survey is **pseudonymised**, which means that no persons can be identified by the answers. *Link: Detailed information on data protection*

Optionally, you can indicate change requests and improvement suggestions for the Charité / UKE at the end of the survey.

Notes

➢ Please read the instructions and the different answer categories carefully.
➢ The questionnaire is divided into different focus areas: *Working environment, professional career, language skills, family and financial situation, life in Germany* as well as *integration and support.*
➢ At the end of the survey, you have the option of answering a few more questions about change requests, suggestions for improvement and possible courses of action for the Charité / UKE as an employer. Answering these questions is voluntary as well.
➢ We would like to take this opportunity to point out that you will be asked questions about possible experiences of discrimination.
➢ We define discrimination as a disadvantage without a justifiable reason, for example on racial or ethnic grounds. By this we refer to the German Federal Anti-Discrimination Agency's (*Antidiskriminierungsstelle des Bundes*) definition of discrimination.[1]

If you have any questions, please contact *Clara Milena Konrad*, *PD Dr. med. Sarah Keller* or *Dr.med. Elif Can.*[2]

Sincerely thanks!

- I would like to take part in the survey and have read and taken note of the information on the content and procedure of the study, the voluntary nature of my participation and data protection.
  Link: Start participation

- I do not wish to participate in the survey.
  Link: Exit questionnaire and leave page

[1] For further information by the German Federal Anti-Discrimination Agency (*Antidiskriminierungsstelle des Bundes*) please click *here.* (https://www.antidiskriminierungsstelle.de/SharedDocs/FAQs/EN/1_what_is_discirmination.html?nn=6580700)

[2] Contact details: Clara Milena Konrad, student of human medicine clara-milena.konrad@charite.de
Private lecturer. Dr. med. Sarah Keller sarah.keller@charite.de
Dr. med. Elif Can, M.A. elif.can@charite.de

Job satisfaction

1. How satisfied are you with your current employment?

Very satisfied

Satisfied

Part/part

Not satisfied

Very dissatisfied

Not specified

2. How satisfied are you with your working hours?

Very satisfied

Satisfied

Part/part

Not satisfied

Very dissatisfied

Not specified

3. How satisfied are you with your payment?

Very satisfied

Satisfied

Part/part

Not satisfied

Very dissatisfied

Not specified

4. How satisfied are you with your working environment?

Very satisfied

Satisfied

Part/part

Not satisfied

Very dissatisfied

Not specified

5. How satisfied are you with the distribution of tasks in your team?

Very satisfied

Satisfied

Part/part

Not satisfied

Very dissatisfied

Not specified

To which extend did the following sentences apply to you *within the past 6 months*?

6. My opinion is respected and appreciated by colleagues.

Not true

Rather not true

More likely to be true

True

Not specified

7. Colleagues appreciate my work performance.

Not true

Rather not true

More likely to be true

True

Not specified

8. Patients appreciate my work performance.

Not true

Rather not true

More likely to be true

True

Not specified

9. Superiors appreciate my work performance.

Not true

Rather not true

Part/part

More likely to be true

True

Not specified

10. I have the feeling of having to prove myself in front of colleagues.

Not true

Rather not true

More likely to be true

True

Not specified

11. I have the feeling of having to prove myself in front of patients.

Not true

Rather not true

More likely to be true

True

Not specified

12. Have you experienced discrimination by colleagues at the same professional level during the past 6 months?

➢ We define discrimination as a disadvantage without a justifiable reason, for example on racial or ethnic grounds. By this we refer to the German Federal Anti-Discrimination Agency's (*Antidiskriminierungsstelle des Bundes*) definition of discrimination.[3]

➢ Optionally, you can use the comment field to note how this discrimination manifested itself.

Yes

No

Not specified

a. If yes: What was the discrimination based on? (Multiple answers possible)

➢ **Discriminatory behaviour can be based on different assumptions, prejudices and apparent reasons. You may indicate several forms of discrimination.**

Age

Disability or chronic disease

Economic/social class

Language

Name

---

[3] For further information by the German Federal Anti-Discrimination Agency (*Antidiskriminierungsstelle des Bundes*) please click *here.* (https://www.antidiskriminierungsstelle.de/SharedDocs/FAQs/EN/1_what_is_discirmination.html?nn=6580700)

Nationality

Physical appearance

Race or ethnicity

Religious beliefs

Sex/Gender

Sexual orientation

Other: *Open entry*

Not specified

UKE: Have you experienced discrimination based on your migration background by colleagues at the same professional level during the past 6 months?

Yes

No

Not specified

13. **CHARITÉ: Have you experienced discrimination by colleagues from other professions during the past 6 months?**

Yes

No

Not specified

   a. ***If yes:*** **What was the discrimination based on? *(Multiple answers possible)***

      Age

      Disability or chronic disease

      Economic/social class

      Language

      Name

      Nationality

      Physical appearance

      Race or ethnicity

      Religious beliefs

      Sex/Gender

      Sexual orientation

      Other: *Open entry*

Not specified

14. **CHARITÉ: Have you experienced discrimination by superiors during the past 6 months?**

Yes

No

Not specified

b. *If yes:* **What was the discrimination based on?** ***(Multiple answers possible)***

Age

Disability or chronic disease

Economic/social class

Language

Name

Nationality

Physical appearance

Race or ethnicity

Religious beliefs

Sex/Gender

Sexual orientation

Other: *Open entry*

Not specified

UKE: Have you experienced discrimination based on your migration background by superiors during the past 6 months?

Yes

No

Not specified

15. **CHARITÉ: Have you experienced discrimination by patients during the past 6 months?**

Yes

No

Not specified

c. *If yes:* **What was the discrimination based on?** ***(Multiple answers possible)***

Age

Disability or chronic disease

Economic/social class

Language

Name

Nationality

Physical appearance

Race or ethnicity

Religious beliefs

Sex/Gender

Sexual orientation

Other: *Open entry*

Not specified

UKE: Have you experienced discrimination based on your migration background by patients during the past 6 months?

Yes

No

Not specified

16. **CHARITÉ: Impact of experiences of discrimination**

Please tick the extent to which the following sentences applied to you *within the last 6 months*:

I have less self-confidence at work due to the experiences of discrimination mentioned above.

Not true

Rather not true

Part/part

More likely to be true

True

Not specified

My professional performance is poorer due to the above-mentioned experiences of discrimination.

Not true

Rather not true

Part/part

More likely to be true

True

Not specified

17. **Do you think that as an employee of the Charité / UKE you are treated equally to your German colleagues? With regard to…**

    a. **Your contract (time limit)?**

       Yes

       No

       Not specified

    b. **Your weekly working hours?**

       Yes

       No

       Not specified

    c. **Your working times (night and weekend shifts)?**

       Yes

       No

       Not specified

    d. **Your payment?**

       Yes

       No

       Not specified

    e. **The distribution of tasks within your team?**

       Yes

       No

       Not specified

    f. **The appreciation of your work performance by *colleagues*?**

       Yes

       No

       Not specified

    g. **The appreciation of your work performance by *superiors*?**

       Yes

       No

Not specified

h. The appreciation of your work performance by *patients*?

Yes

No

Not specified

i. Opportunities for further training and promotion?

Yes

No

Not specified

j. The opportunity to express your opinion in discussions among colleagues?

Yes

No

Not specified

Professional career

1. **Since when have you been working at Charité / UKE?**

   Year

2. **Profession/field of work**

   Nursing and health care

   Medical assistant or technical assistant (e.g. anaesthesia, surgery, laboratory, radiology)

   Scientific staff, medical

   Scientific staff, non-medical

   Other staff

   Not specified

   a. ***In case of "scientific staff, medical":***

      Assistant doctor

      Specialist doctor

      Researcher

      Other: *Open entry*

      Not specified

   b. ***In case of "scientific staff, non-medical":***

      PhD student

      Post-doc

      Professor/Habilitated

      Other: *Open entry*

      Not specified

   c. ***In case of any except "scientific staff, medical":*** **Which qualification did you obtain?**

      University degree (Bachelor)

      University degree (Master)

      Diploma

      Vocational training

      Other: *Open entry*

      Not specified

3. **Which hierarchical level are you working on?**

Leading function

Non-leading function

Not specified

4. **Are you working part time?**

No

Yes

Not specified

5. **Do you have a temporary working contract?**

Yes

No

Not specified

6. **Did you take part in a guided programme for your employment in Germany? For example Triple Win Programme by the GIZ or a scholarship program of the Charité / UKE and your home country?**

Yes

No

Not specified

UKE: Did you participate in the program "Anpassungsqualifizierung" ("Adjustment Qualification") of the UKE Academy for Education and Career to have your qualifications recognised?

Yes

No

Not specified

7. **Please tick as appropriate:**

I completed my vocational training / studies abroad before coming to Germany.

I completed my vocational training / studies in Germany.

Other: *Open entry*

Not specified

In case of option 1: "after completion of vocational training/studies":

Please specify to what extent the following sentences apply to you:

a. **My job qualifications were recognised without any problems.**

Not true

Rather not true

Part/part

More likely to be true

True

Not specified

b. **I was well informed about the recognition procedure.**

Not true

Rather not true

Part/part

More likely to be true

True

Not specified

c. **I felt discriminated against by employees of the authorities.**

Not true

Rather not true

Part/part

More likely to be true

True

Not specified

d. **Please tick as appropriate:**

My current professional activity is below my qualification.

My current professional activity is above my qualification.

My current professional activity corresponds to my qualification.

e. **How long did it take for your qualification to be recognised from the time you submitted your documents? (In months)**

Months

f. **Did you have to take an examination in Germany for the recognition of your professional qualifications?**

Yes

No

Not specified

8. **Do you have German citizenship?**

Yes

No

Not specified

a. ***If no:*** **Is your residence status limited?**

Temporary

Permanent

Not specified

Language skills

1. **What is your mother tongue?**

   Open entry

2. **How would you evaluate your current German language skills?**

   Close to mother tongue (C2)

   Competent speech application (C1)

   Good and independent speech application (B1 and B2)

   Basic knowledge (A1 and A2)

   Not specified

3. **Have you already started learning German in your home country?**

   Yes

   No

   Not specified

   a. ***If yes:*** **Where?**

      Public School

      Private school

      University

      Language school (private)

      Other: *open entry*

      Not specified

4. **Were you required to provide proof of German language skills by the German authorities?**

   Yes

   No

   Not specified

   a. ***If yes*****: For which level?**

      Very competent to fluent (C1-2)

      Independent application, good communication (B1-B2)

      Basic knowledge (A1-A2)

      Not specified

5. **Did you attend a language course (regardless of the requirements of the authorities)?**

Yes

No

Not specified

a. ***If yes*: Where?**

In home country

In Germany

Not specified

b. **Did you complete a language course organized by the Charité / UKE?**

Yes

No

Not specified

c. **Did you receive any financial support for your language course?**

Yes

No

Not specified

d. **Was financing the language course challenging for you?**

Yes

No

Not specified

e. **Was the language course specifically designed for your profession?**

Yes

No

Not specified

6. **Do or did you have difficulties in your professional life due to a lack of language skills?**

Yes

No

Not specified

If yes:

a. **Did these difficulties occur *within the last 6 months?***

Yes

No

Not specified

b. **What did you have difficulties with? *Multiple answers possible***

Communication with patients

Communication with colleagues

Documentation

During phone calls

Speaking about problems

Other: *Open entry*

Not specified

7. **Do or did you have the feeling of not being able to fully exercise your professional skills due to a lack of language skills?**

Yes

No

Not specified

a. ***If yes:* During the last 6 months?**

Yes

No

Not specified

8. **Have you been asked to translate for colleagues within the last 6 months?**

Yes

No

Not specified

a. ***If yes:* How often do you translate for your colleagues? Please give a rough estimate.**

Once per day

Once per week

Once per month

Once per year

Less than once per year

Not specified

Family and financial situation

The following information is needed in order to assess the results more accurately. We assure you that your information will be evaluated anonymously and that no conclusions can be drawn to you personally.

1. **Do you have additional old-age insurance or financial security in case you are no longer able to work (e.g. own real estate, private pension insurance, life insurance)?**

   Yes

   No

   Not specified

2. **What is your family situation?**

   Single

   In a partnership

   Married/registered partnership

   Other: Open entry

   Not specified

   In case of options 2 to 4:

   a. **Does your partner live with you in Germany?**

      Yes

      No, abroad elsewhere

      No, in the home country

      Not specified

   b. **Is your partner in employment?**

      Yes

      No

      Not specified

   c. **Do you have to support your partner financially?**

      Yes

      No

      Not specified

3. **Do you have children?**

   Yes

No

Not specified

If yes:

a. **How many?**

Open entry

a. **How old are your children? *Multiple answers possible***

0 to less than 10 years

10 to less than18 years

19 to less than 30 years

over 30 years

Not specified

b. **Do your children live with you in Germany?**

Yes

No, abroad elsewhere

No, in home country

Not specified

4. **Do you have to co-finance other relatives or friends with your income? Think of parents, grandparents or siblings for instance.**

Yes

No

Not specified

If yes:

a. **Who do you have to co-finance additionally? *Multiple answers possible***

Parents

Grandparents

Siblings

Others: Open entry

Not specified

b. **Do the persons co-financed by you live in Germany?**

Yes

No, abroad elsewhere

No, in the home country

Not specified

5. Apart from financial assistance, are you involved in the support or care of relatives / friends?

Yes

No

Not specified

If yes:

a. By who? *Multiple answers possible*

Children

Partner

Parents

Grandparents

Siblings

Others: *Open entry*

Not specified

b. What do you estimate the weekly time required for this? *(stated in hours per week)*

Hours

Life in Germany

1. Which district do you live in?

List of districts

2. Have you felt discriminated against in public places (train, shops, public places) in Germany within the last 6 months?

Yes

No

Not specified

a. *If yes:* **What was the discrimination based on?** ***(Multiple answers possible)***

Age

Disability or chronic disease

Economic/social class

Language

Name

Nationality

Physical appearance

Race or ethnicity

Religious beliefs

Sex/gender

Sexual orientation

Other: *Open entry*

Not specified

3. Have you felt discriminated against in your social environment (e.g. by friends, acquaintances) in Germany within the last 6 months?

Yes

No

Not specified

a. *If yes:* **What was the discrimination based on?** ***(Multiple answers possible)***

Age

Disability or chronic disease

Economic/social class

Language

Name

Nationality

Physical appearance

Race or ethnicity

Religious beliefs

Sex/Gender

Sexual orientation

Other: *Open entry*

Not specified

4. **Do you intend to stay in Germany permanently?**

Yes

No

Not specified

a. ***If no:*** **Where are you planning to move to?**

Home country

Other: *Open entry*

Not specified

Integration and support

Please tick as appropriate:

1. **Through which institutions did you receive support? Please specify how helpful the support was for you.** ***Multiple answers possible***

CHARITÉ: Welcome Centre

Extremely helpful

Very helpful

Somewhat helpful

Only partly helpful

Not helpful at all

Unknown

Commissioner for Integration

Extremely helpful

Very helpful

Somewhat helpful

Only partly helpful

Not helpful at all

Unknown

Human Resources Division

Extremely helpful

Very helpful

Somewhat helpful

Only partly helpful

Not helpful at all

Unknown

Professional and staff council

Extremely helpful

Very helpful

Somewhat helpful

Only partly helpful

Not helpful at all

Unknown

Trade Union

Extremely helpful

Very helpful

Somewhat helpful

Only partly helpful

Not helpful at all

Unknown

Others: *Open entry*

Extremely helpful

Very helpful

Somewhat helpful

Only partly helpful

Not helpful at all

Unknown

None

2. By whom did you receive additional support? ***Multiple answers possible***

Colleagues

Supervisors

Family

Friends and acquaintances

None

Other: *Open entry*

3. What were you supported in? ***Multiple answers possible***

Formalities (work contract, recognition of qualifications, residence permit)

Administrative procedures

Search of accommodation

Mobility/transportation

Childcare

School and education of children

Acquisition of language skills

Job induction training

Vocational education and further training

Social life/everyday life

Other: *Open entry*

4. What else helped you during the process of integration?

Translation programmes

Social networks/online communities

Networking with other international colleagues

Private communities/municipalities

Nothing

Other: *Open entry*

5. For what would you have wished to receive further support? *Multiple answers possible*

Formalities (work contract, recognition of qualifications, residence permit)

Administrative procedures

Search of accommodation

Mobility/transportation

Childcare

School and education of children

Acquisition of language skills

Job induction training

Vocational education and further training

Social life/everyday life

Other: *Open entry*

General questions

1. **Please indicate your biological sex**

   Female

   Male

   Diverse / Other

   Not specified

2. **What year were you born?**

   Year

3. What country were you born in?

Open Entry

4. Since when are you living in Germany (year)?

Year

5. Do you have any comments on the questions asked or on the information you have provided?

Thank you very much for your participation!

If you wish, you can now answer a few questions about possible courses of action for the Charité / UKE as an employer.

If you want to finish the questionnaire, click on "Submit".

If you are willing to formulate concrete suggestions, click on "Answer further questions".

- **Submit**
- **Answer further questions**

Part 2: Optional questions after completion of the questionnaire

---

1. **Do you have an idea what the Charité / UKE can do for you to feel comfortable at your workplace?**

   Open entry

2. **Do you have an idea what the Charité / UKE can do for you to feel comfortable in Berlin / Hamburg?**

   Open entry

3. **What could be learned from other international institutions? Please name the institution and your suggestions!**

   Open entry

4. **Would you be interested in networking with international colleagues in this institution?**

   Yes

   No

   a. *If yes:* **What do you think such networking could look like? Please make suggestions!**

      Open entry

5. **Do you have any other wishes or suggestions for improvement? Please name them!**

   Open entry

6. **Do you have any comments on the questions asked or on the information you have provided?**

   Open entry

Thank you for answering these further questions!

Please click now on "Submit".

- **Submit**

Thank you very much for your participation!
